# Vascular surgery in the most populous state in the Amazon: socio-professional profile and aspirations of the specialty

**DOI:** 10.1590/1677-5449.210039

**Published:** 2021-06-16

**Authors:** José Maciel Caldas dos Reis, Deivid Ramos dos Santos, Inez Ohashi Torres, Nelson De Luccia

**Affiliations:** 1 Universidade do Estado do Pará – UEPA, Belém, PA, Brasil.; 2 Centro Universitário Metropolitano da Amazônia – UNIFAMAZ, Belém, PA, Brasil.; 3 Universidade de São Paulo – USP, Faculdade de Medicina, Hospital das Clínicas, São Paulo, SP, Brasil.

**Keywords:** medical specialties, vascular surgery, physician distribution, demographic data, especialidades médicas, cirurgia vascular, distribuição de médicos, dados demográficos

## Abstract

**Background:**

There is a dearth of studies conducted to understand the socio-professional profile of the vascular surgery specialty and the population demands of specific regions, which are needed to support creation of care policies and direct infrastructure improvements in healthcare.

**Objectives:**

The purpose of this study was to describe the socio-professional profile of vascular surgeons in the state of Pará, Brazil, to guide creation of tools for professional improvement.

**Methods:**

A cross-sectional, self-report survey was conducted in Pará using a questionnaire comprising 30 questions covering six main topics.

**Results:**

All vascular surgeons actively practicing in the state participated in this study. The total number of specialists was 59, with 71.2% working in the greater Belém area and 16.9% exclusively practicing in the interior of the state. The mean age of these professionals was 48 ± 11.1 years, 86.4% of respondents were men, 64.4% of surgeons had completed medical residency, and 96.6% (n=57) of the surgeons would like to improve their skills in venous surgery, echo-guided vascular access, and endovascular surgery. The method of professional improvement of greatest interest was simulation courses (hands-on), endorsed by 93% of the participants.

**Conclusions:**

Pará has 59 vascular surgeons. These professionals mainly work in the greater Belém (71.2%), in hospitals (100%) or in private clinics or offices (94.9%), performing a wide range of procedures, including venous and arterial surgery, amputations, and provision of hemodialysis access. More than 90% of these surgeons were satisfied professionally and reported that they would choose the specialty again. However, 22% had a pessimistic view of the specialty’s future. The vast majority of professionals (96.6%) consider that training or a continuing education program are necessary.

## Resumo

**Contexto:**

Há uma falta de estudos necessários para entender o perfil socioprofissional da especialidade e as demandas específicas da população de uma região específica, a fim de subsidiar a criação de políticas assistenciais e a melhoria na infraestrutura da assistência à saúde.

**Objetivos:**

O objetivo deste estudo foi descrever o perfil socioprofissional de cirurgiões vasculares no Pará para orientar a criação de ferramentas de melhoria profissional.

**Métodos:**

Foi realizado um levantamento transversal no Pará utilizando um questionário com 30 questões que envolvia seis temas principais.

**Resultados:**

Todos os cirurgiões vasculares ativos participaram deste estudo. O número total de especialistas foi de 59, dos quais 71,2% trabalhavam na grande Belém e 16,9% exclusivamente no interior do estado. A idade média dos profissionais foi de 48 ± 11,1 anos, e 86,4% dos entrevistados eram homens. Além disso, 64,4% dos cirurgiões haviam completado a residência médica, e 96,6% (n = 57) deles gostariam de ter melhorias em cirurgia venosa, acesso vascular ecoguiado e cirurgia endovascular. As áreas de maior interesse para melhorias são cursos de simulação (práticos), com 93% dos participantes interessados.

**Conclusões:**

O Pará tem 59 cirurgiões vasculares, os quais trabalham principalmente na grande Belém (71,2%) em hospitais (100%) ou em clínicas privadas ou consultórios (94,9%) e realizam uma ampla gama de procedimentos, incluindo cirurgias venosas e arteriais, amputações e acessos para hemodiálise. Mais de 90% dos cirurgiões estavam satisfeitos e relataram que escolheriam a especialidade novamente; entretanto, 22% tinham uma visão pessimista do futuro da especialidade. A grande maioria dos profissionais (96,6%) considera a necessidade de qualificação ou de um programa de educação continuada.

## INTRODUCTION

Over the years, the Brazilian Ministry of Health has been encouraging doctors to work in rural areas.^1^ However, the unequal distribution of specialist doctors across the national territory remains an important issue.^1^^,^^2^ A lack of technical and educational infrastructure discourages new specialists from leaving large urban centers to move to provincial areas of states.^2^^,^^3^

The state of Pará is the second largest in Brazil, with an area equivalent to Peru and twice the size of France. It is considered the most populous state in the Brazilian Amazon, with approximately 8,690,000 inhabitants, according to the last census in 2020.^4^ The state’s vast size hinders uniform distribution of professionals and constitutes a barrier to access to specialized healthcare for populations living in regions far from the capital.

The process of training healthcare specialists and their professional ingress into the labor market is complex, multifactorial, and dynamic. Therefore, there is a need to conduct studies to understand the socio-professional profile of the specialties and the population demands of specific regions, to support creation of care policies and guide healthcare infrastructure improvements.^4^^-^7

However, despite the great importance of the subject, there are few studies investigating the geographical distribution of vascular surgeons in the states of the Amazon and issues related to specialists’ aspirations, such as areas of activity of greatest interest and desire for professional improvement. Thus, efforts should be made to compile information of relevance to new specialists in the field, to the Brazilian Society of Angiology and Vascular Surgery (SBACV; official entity dedicated to the study and practice of vascular surgery in the country), and to the government.

The objective of this article is to describe the socio-professional profile of vascular surgeons in Pará, to guide the creation of tools for professional improvement.

## METHODS

A cross-sectional study was conducted using a questionnaire. This study was approved by the Research Ethics Committee at the Centro Universitário Metropolitano da Amazônia (CAAE: 14947919.5.0000.5701), under ruling number 3,498,631. Written informed consent was obtained from all participants, and the anonymity of all participants was ensured.

The study included all active vascular surgeons in the state of Pará. The exclusion criteria were vascular surgeons currently inactive, retired, or practicing the specialty without specialist training.

The questionnaire comprised 30 questions covering the following six main topics: sociodemographic aspects, professional experience and training, scientific management and involvement, employment status, professional improvement, and satisfaction with the specialty.

The questionnaires were distributed in person and by e-mail to all specialists in the state of Pará. Distribution was completed by December 2019 and respondents were allowed a maximum period of 30 days after receipt of questionnaires to answer and return them.

Categorical variables were analyzed using BioEstat® 5.3 software. The significance level was established at α=0.05 and significant values were indicated with an asterisk (*). Quantitative variables are presented as means and standard deviations (mean ± standard deviation). Nominal qualitative variables are presented in absolute values and percentages.

## RESULTS

All vascular surgeons actively practicing in the state of Pará up to the end of 2019 participated in this study. The total number of specialists was 59, of whom 71.2% were working in the greater Belém area, 16.9% were working exclusively in the interior of the state, and 11.9% were working in the capital and interior of the state.

The mean age of the professionals was 48 ± 11.1 years. The youngest vascular surgeon was 30 years old and the oldest still practicing was 72 years old. As for sex, 86.4% of respondents were men.

The analysis of professional training showed that 64.4% of surgeons had completed medical residency, 27.1% had completed an internship recognized by the SBACV, and only 8.5% had done unrecognized internships in the specialty. The mean time elapsed since completion of specialization for these professionals was 17.2 ± 11.7 years. It was found that 25.4% (n=15) of the professionals were engaged in academic activities at medical schools in the state, mostly (60%) at the Federal University of Pará. However, only 15.3% of the professionals had postgraduate qualifications, 88.9% of whom had a Master’s degree, 44.4% a Doctoral degree, and 11.1% a Master of Business Administration degree. Most professionals (66.1%) had an SBACV qualification.

With regard to the predominant practice settings, 100% provided care at hospitals and 94.9% also practiced in clinics or offices. With respect to the participants’ area of expertise within the specialty, the full range of vascular surgery activities were represented, as shown in detail in Tables 1 and 2.

**Table 1:** Areas of expertise of vascular surgeons in the state of Pará, Brazil.

**Areas of expertise within the specialty**	**n**	**% (n=59)**
Vascular surgery	58	98.3%
Angiology	45	76.3%
Endovascular surgery	26	44.1%
Vascular ultrasound with Doppler	25	42.4%
Interventional radiology	5	8.5%

**Table 2:** Types of surgeries performed in hospital settings by the vascular surgeons in the state of Pará, Brazil.

**Hospital activity**	**n**	**% (n=59)**	**Time dedicated (%)** *
			Mean ± standard deviation
Venous surgery	54	91.5%	48.7 ± 24.2
Amputations and debridement	52	88.1%	19.7 ± 14.9
Arterial surgery	44	74.6%	10.9 ± 9.2
Hemodialysis or chemotherapy access	41	69.5%	13.2 ± 12.5
Emergency/trauma surgery	33	55.9%	7.7 ± 11.9

* Estimated percentage of time dedicated to performing each type of surgery.

Regarding membership of medical societies, 74.6% of the specialists reported being a member of a medical society, most of whom (93.2%) were members of the SBACV (Pará state chapter). As for management activities in medical societies or hospitals, 17 specialists (28.8%) were involved in such activities, with 82.4% of these involved in the SBACV (Pará state chapter).

A majority of the specialists (72.7%) reported working in a shifts system. However, some specialists also worked in other areas, such as general surgery (13.6%), intensive care (13.6%), and even emergency care (4.5%) in the state. Additionally, 20.3% (n=12) of these surgeons had other non-medical sources of income.

When asked about professional development, 96.6% (n=57) of the surgeons replied that they would like to improve in some aspect of the profession and the most frequently endorsed areas of professional improvement among the options provided were venous surgery, echo-guided vascular access, and endovascular surgery (p<0.0001; G-test of goodness-of-fit).

The areas of improvement in which most participants were interested are shown in Table 3, and the preferred method for improvement was simulation courses (hands-on), in which 93% of the participants were interested.

**Table 3:** Areas in which vascular surgeons in the state of Pará were interested in improving.

**Areas of interest**	**n**	**Frequency**	**%**
**Open arterial surgery**	**9**		
Carotid		7	77.8%
Aorta		7	77.8%
Limbs		9	100.0%
**Endovascular surgery**	**30**		
Carotid		25	83.3%
Aorta		28	93.3%
Limbs		28	93.3%
**Trauma surgery**	**8**		
Aorta		7	87.5%
Limbs		8	100.0%
**Venous surgery**	**52**		
Radiofrequency		49	94.2%
Laser		50	96.2%
Foam		48	92.3%
**Vascular access**	**31**		
Echo-guided access		31	100.0%
Arteriovenous fistula		11	35.5%

Table 4 summarizes the responses to questions about participants’ satisfaction with their specialty.

**Table 4:** Satisfaction of the vascular surgeons in the state of Pará with their specialty.

**Satisfaction with the specialty**	**Frequency**	**% (n=59)**
**Professionally satisfied**		
Yes*	55	93.2%
No	4	6.8%
**Would you opt for vascular surgery again**		
Yes*	56	94.9%
No	3	5.1%
**Opinion on the future of the specialty**		
Optimistic view**	46	78.0%
Pessimistic view	13	22.0%

* p<0.0001; G-test

** p<0.0001; chi-square test

**Figure 1:**
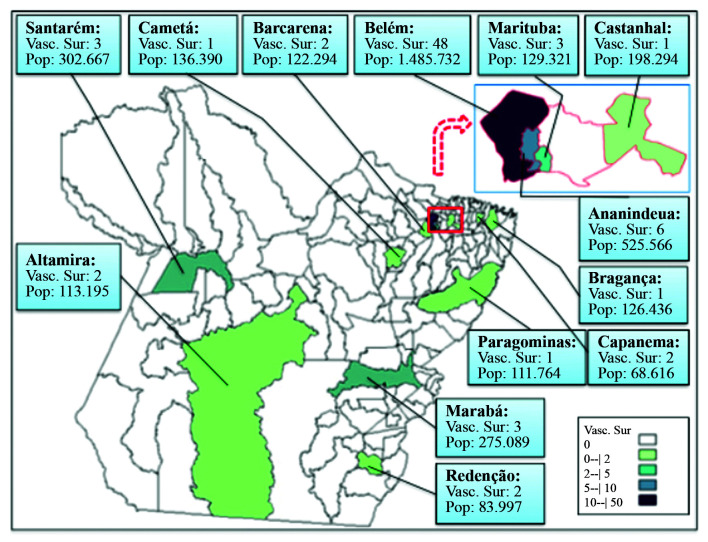
Illustrates the geographical distribution of vascular surgeons by municipal districts in the state of Pará up to the end of 2019. Source: Research protocol and IBGE 2019.

Figure 1 illustrates the geographical distribution of vascular surgeons within the state of Pará, by municipal district, up to the end of 2019.

## DISCUSSION

Since the revolution that has occurred in the field of vascular surgery over recent years, it has become a great challenge for both surgeons in training and experienced professionals to keep up-to-date on the various open and endovascular procedures. The first step in developing an up-to-date and continuous education program is to understand the situation and needs of each location. Hence, the elaborate questionnaire used in this study considered the situation and concerns of vascular surgeons in the state of Pará.

Research into the socio-professional profile of specialist surgeons using questionnaires reports varying percentages of participation.^5^^,^^6^ In this study, the active search for members of the specialty resulted in participation by 100% of the professionals working in the state, and therefore, the study portrays the true situation of the specialty in the most populous state in the Amazon.^4^ There are currently 59 professionals practicing in the state, mostly men (63.7%), with a mean age of 48 years (63.7%).

The distribution of vascular surgeons followed the population distribution of the region, that is, 71.2% of professionals concentrated their activities in the greater Belém area, which is equivalent to a ratio of one specialist per 59.5 thousand inhabitants. The national mean is 2.07 specialists for every 100 thousand inhabitants.^1^ This deficit in professionals is partly because of the fact that Pará has no vascular surgery residency programs yet, although it already has six Ministry of Education accredited residency programs in general surgery in the state capital alone.

Pará had 9,212 doctors registered at the end of 2020^1^ and, according to the Brazilian Institute of Geography and Statistics (IBGE), Belém has a population of 1,499,641 inhabitants, with a total of 42 vascular surgeons working for an estimated population of approximately 2.5 million inhabitants in the greater Belém area (the metropolitan region of the state).^4^ Therefore, the ratio of vascular surgeon to inhabitants is 2.8 specialists for every 100 thousand inhabitants in Belém, while in the metropolitan region this ratio decreases to 1.6 specialists for every 100 thousand inhabitants (the mean in the state is 0.5 to 0.8 for each 100 thousand inhabitants). The national mean is 2.07 for every 100 thousand inhabitants.^1^ The World Health Organization recommends one specialist per 17 thousand inhabitants in developed countries, while in developing countries, such as Brazil, a ratio of up to one vascular surgeon per 35 thousand inhabitants is acceptable.^6^^,^^7^ In the northern region of Brazil, the 2018 vascular census reported a proportion of one specialist for every 180,561 inhabitants.^8^

According to Scheffer et al.,^1^ Brazil is going through demographic and epidemiological changes, with a strong trend towards population aging and an increasing prevalence of chronic diseases.^1^ This will require a reorganization of health services to address increased health demands and the population’s needs. However, the vast majority of the population has no access to vascular treatment and the distribution of professionals is not uniform, since vascular surgeons prefer large urban centers for their clinical practice.^9^^-^11

Vascular surgeons work in clinics or offices and public and private hospitals, with an estimated proportion of 42.8% of their time dedicated to clinics and offices, 29.5% to public hospitals, and 27.9% to private hospitals. Furthermore, there are multiple choices and specialists usually work concurrently in more than one location or with different employment relationships.

Regarding professional improvement, 74.6% (n=44) of the specialists reported seeking support from scientific societies, which supported their continuous education, and being constantly encouraged to join the SBACV, which in Pará accounted for 93.2% of those affiliated to a medical society.

To the best of our knowledge, this is the first study to analyze the socio-professional profile of vascular surgeons in the state of Pará. The finding that 71.2% of the professionals practiced in the greater Belém area illustrated the growing trend toward urban centralization of specialized services. Thus, large metropolitan hospitals with technology, equipment, and human resources attract more specialists, since vascular surgery and its areas of activity are directly related to socio-economic conditions, public policies, and infrastructure.^6^^,^^7^^,^^9^^-^12

However, since 16.9% of the specialists work exclusively in the interior of the state, healthcare policies should provide the infrastructure conditions to enable the full range of vascular surgery procedures to be fully performed even in remote municipalities.

Thus, attracting and retaining specialists to work in the interior of the state continues to be a permanent challenge for several reasons, even in developed countries^3^^,^^13^^-^15 O’Sullivan et al.^3^ surveyed 3,479 physicians from metropolitan and regional locations in Australia, reporting no difference in overall job satisfaction. However, a survey of 137 surgeons working in Australian towns with populations of less than 50,000 showed that the main challenges identified included working shifts, professional isolation, lack of privacy, and their children’s education.^3^ Thus, a complete understanding of the needs of the specialist and specialty is essential to retaining professionals in the interior of states and to establishing an effective surgical practice.

Incorporation of new technologies in the various areas of vascular surgery has created the need for constant additional training for specialists, either to complete their education or to improve it.^5^^,^^16^^,^^17^ Thus, in addition to technical knowledge, specialists seek essential skills, competences, and behaviors to deal with the challenges of the specialty and new technologies.^10^^-^12 Hands-on simulation courses were the option chosen by 93% of the specialists in the state, indicating a growing trend towards using realistic simulation technologies. The areas of greatest interest for improvement were venous surgery (n=52), echo-guided vascular access (n=31), and endovascular surgery (n=30). Thus, it is important that these professionals are constantly developing, learning, and accumulating new knowledge, and the information and skills acquired can have an impact on social insertion within the scope of healthcare.^6^^,^^9^^-^11

Satisfaction with the specialty was positive, with 93.2% stating that they were professionally satisfied and 94.9% stating that they would choose vascular surgery again, although a need for concerted action by the specialty was also indicated, since the pessimistic view endorsed by 22% of the interviewees is directly related to dissatisfaction with and deterioration of the relationship with health insurance providers and insurers, compounded by invasion of the specialty by tentacles from other entities.

An important aspect to be considered is the lack of vascular surgeons that some countries have reported, as shown by Berger and Mace^5^ in an article in which they expressed their concern regarding a shortage of vascular surgeons in the future.

This study presents important observations; however, it has some limitations, since it was a cross-sectional study that involved only one of Brazil’s states. Despite this, there is still a scarcity in the Brazilian literature of published studies conducted with similar methods, holding up a mirror to the specialty in a given area. This study has the potential to be extended to all the states in Brazil and to other medical specialties, enabling creation of specific training policies that are adequate to the needs of each region, since demographic characteristics and infrastructure conditions influence the centralization of specialists.

## CONCLUSIONS

Currently, the state of Pará has 59 vascular surgeons, with a mean age of 48 ± 11.1 years, and a predominance of male professionals (86%). These professionals work mainly in the greater Belém (71.2%), in hospitals (100%) or in private clinics or offices (94.9%), performing a wide range of procedures, including venous and arterial surgery, amputations, and provision of hemodialysis access. More than 90% of these surgeons were satisfied and reported that they would choose the specialty again. However, 22% had a pessimistic view of the specialty’s future. The vast majority of professionals (96.6%) considered there was a need for training or a continuing education program.
